# Cerebellar tDCS: A Novel Approach to Augment Language Treatment Post-stroke

**DOI:** 10.3389/fnhum.2016.00695

**Published:** 2017-01-12

**Authors:** Rajani Sebastian, Sadhvi Saxena, Kyrana Tsapkini, Andreia V. Faria, Charltien Long, Amy Wright, Cameron Davis, Donna C. Tippett, Antonios P. Mourdoukoutas, Marom Bikson, Pablo Celnik, Argye E. Hillis

**Affiliations:** ^1^Department of Neurology, Johns Hopkins University School of MedicineBaltimore, MD, USA; ^2^Department of Radiology, Johns Hopkins University School of MedicineBaltimore, MD, USA; ^3^Department of Otolaryngology—Head and Neck Surgery, Johns Hopkins University School of MedicineBaltimore, MD, USA; ^4^Department of Biomedical Engineering, The City College of New York of CUNYNew York, NY, USA; ^5^Department of Physical Medicine and Rehabilitation, Johns Hopkins University School of MedicineBaltimore, MD, USA; ^6^Department of Neuroscience, Johns Hopkins University School of MedicineBaltimore, MD, USA; ^7^Department of Cognitive Science, Johns Hopkins UniversityBaltimore, MD, USA

**Keywords:** cerebellar tDCS, stroke, aphasia, spelling therapy, resting state fMRI

## Abstract

People with post-stroke aphasia may have some degree of chronic deficit for which current rehabilitative treatments are variably effective. Accumulating evidence suggests that transcranial direct current stimulation (tDCS) may be useful for enhancing the effects of behavioral aphasia treatment. However, it remains unclear which brain regions should be stimulated to optimize effects on language recovery. Here, we report on the therapeutic potential of right cerebellar tDCS in augmenting language recovery in SMY, who sustained bilateral MCA infarct resulting in aphasia and anarthria. We investigated the effects of 15 sessions of anodal cerebellar tDCS coupled with spelling therapy using a randomized, double-blind, sham controlled within-subject crossover trial. We also investigated changes in functional connectivity using resting state functional magnetic resonance imaging before and 2 months post-treatment. Both anodal and sham treatments resulted in improved spelling to dictation for trained and untrained words immediately after and 2 months post-treatment. However, there was greater improvement with tDCS than with sham, especially for untrained words. Further, generalization to written picture naming was only noted during tDCS but not with sham. The resting state functional connectivity data indicate that improvement in spelling was accompanied by an increase in cerebro-cerebellar network connectivity. These results highlight the therapeutic potential of right cerebellar tDCS to augment spelling therapy in an individual with large bilateral chronic strokes.

## Introduction

Aphasia is a leading cause of disability following stroke and can affect every aspect of daily life, including interpersonal relationships, work, and community interactions. Speech-language therapy is the mainstay of treatment. Therapy is beneficial for language recovery; however, gains in therapy are variable and progress may be slow, especially after large, chronic left hemisphere lesions (Brady et al., 2016). Recently, neuromodulation with tDCS has been introduced to increase the efficiency of speech and language therapy (for recent reviews see de Aguiar et al., 2015; Sebastian et al., 2016b). Studies indicate that anodal tDCS over peri-lesional left hemisphere (LH) language regions has the potential to augment language outcomes in individuals with chronic aphasia (e.g., Baker et al., 2010; Fiori et al., 2011; Fridriksson et al., 2011; Vestito et al., 2014). However, large LH stroke impedes improvement of language functions that are dependent on LH networks. In such cases, enhancing the function of non-damaged hemisphere with the goal of facilitating compensation has been investigated. However, some data suggest that recruitment of right hemisphere (RH) regions can be maladaptive in the chronic stage. Also, several studies have shown benefit of RH inhibitory (cathodal) tDCS or combined LH anodal tDCS + RH cathodal tDCS (e.g., Marangolo et al., 2014; Manenti et al., 2015). However, inhibition of the RH might have detrimental effects on cognitive functions that normally rely on the RH. Previous studies have not evaluated the effect of tDCS in individuals with large, bilateral chronic stroke.

This case study illustrates the potential usefulness of a novel electrode placement for tDCS augmentation of language therapy in chronic post-stroke aphasia: the right cerebellum.

Evidence from functional neuroimaging and neuroanatomical investigations indicate that the right cerebellum is important for language and cognitive functions (e.g., Leiner et al., 1989; Schmahmann, 1991, 2001; Middleton and Strick, 1994; Stoodley and Schmahmann, 2009; Murdoch, 2010; Stoodley et al., 2012; Marien et al., 2014; for recent reviews see De Smet et al., 2013; Keren-Happuch et al., 2014). Damage to the right cerebellum has been associated with deficits in a variety of language tasks (e.g., Hassid, 1995; Marien et al., 1996, 2000; Gómez Beldarrain et al., 1997; Fabbro et al., 2004; Baillieux et al., 2010). In addition, cerebellar tDCS studies in healthy individuals provide evidence that right cerebellar tDCS modulates cognitive and language functions such as verb generation (Pope and Miall, 2012), verbal fluency (Turkeltaub et al., 2016), working memory (Boehringer et al., 2013; Macher et al., 2014), and implicit learning (Ferrucci et al., 2013). See Grimaldi et al. (2016) for a recent review. Beneficial cognitive effects from right cerebellar tDCS have been found for both anodal and cathodal stimulation.

Given the role of the cognitive and language functions of the cerebellum and the ability of cerebellar tDCS to modify behavior in healthy individuals, cerebellar tDCS may have a uniquely valuable therapeutic role for individuals with aphasia. Furthermore, cerebellum can be stimulated even in patients with aphasia associated with bilateral hemispheric strokes. In addition, the cerebellum is regarded as an important region involved in skill learning (Morton and Bastian, 2006; Galea et al., 2011). Therefore, cerebellar tDCS could also augment response to language therapy by enhancing learning skills.

Here, we report behavioral and neural effects of right cerebellar tDCS with behavioral spelling treatment in a participant who sustained bilateral MCA infarct resulting in aphasia and complete anarthria. Participant SMY is mute following his second stroke but has retained some ability to write and type. Because he depends on writing to communicate, recognizable spelling is critical for effective social function. Therefore, cerebellar tDCS plus behavioral spelling treatment could improve spelling recovery through its roles in language and learning. We sought to evaluate the following hypotheses: (1) Improvement in spelling to dictation (in treated and untreated words) will be greater with tDCS + spelling treatment than with sham + spelling treatment; (2) Improvement will last longer after tDCS treatment than sham treatment at 2 months post-treatment; (3) Improvement in other language tasks (written picture naming) will be greater after tDCS than sham; (4) Functional connectivity between the right cerebellum and the residual left and right hemisphere language regions of interest will be greater post-treatment compared to pre-treatment.

## Case report

### Patient history

**Figure 1:**
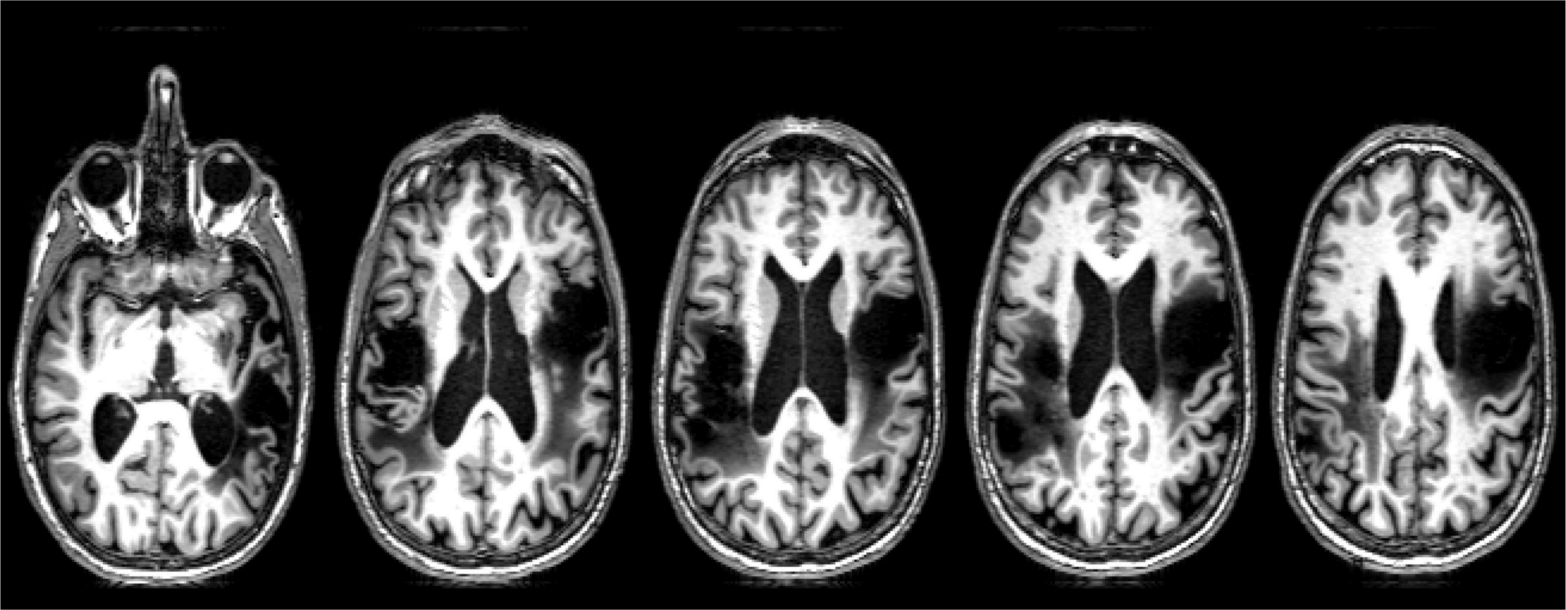
**Lesion map of SMY**.

SMY is a 57-year-old, right-handed man with a master's degree, employed as an architect until he had an ischemic stroke due to carotid dissection. MRI revealed left MCA territory infarct, involving frontal, temporal, and insular cortex. This stroke resulted in right hemiparesis and aphasia. He underwent extensive inpatient and outpatient rehabilitation, and showed resolution of hemiparesis and substantial improvement in language. He survived a second (right hemisphere) stroke due to carotid dissection 4 years later. MRI revealed acute infarct of right MCA territory, involving the fronto-parietal and insular cortex (Figure 1). His second stroke resulted in left hemiparesis, dysphagia necessitating PEG placement, aphasia, and no speech production due to anarthria. Please see Figure 1 for lesion location.

He was enrolled in the study in 2015, 5 years after his second stroke. At the time of his enrollment, SMY was independent in activities of daily living and resided with his wife. SMY scored 26/28 on the auditory comprehension subtest on the Aphasia Diagnostic Profile test (Helm-Estabrooks, 1992). Participant SMY is mute and communicates by writing in a book or on an iPad, augmented with a variety of gestures. This study was carried out in accordance with the recommendations of the ‘Johns Hopkins Medicine Institutional Review Boards’ with written informed consent from all subjects. SMY gave written informed consent in accordance with the Declaration of Helsinki.

### Spelling and naming performance

Given that SMY is mute and the focus of treatment was on spelling, only written language was assessed in detail. SMY's narrative writing consisted of simple sentences with frequent phonologically implausible nonword errors (center → cect), semantic errors (garage → house), and letter omissions (piano → pian). SMY was able to write simple and common 3-letter words and some 4-letter words without difficulty. He was not able to identify his errors in writing. The Johns Hopkins Dysgraphia Battery (Goodman and Caramazza, 1985) was administered (See Supplementary Material).

Spelling to dictation of words and pseudowords was very impaired (12% accurate on words and 0% accurate on pseudowords). On words, he showed a significant effect of grammatical word class (e.g., verbs vs. nouns 36 vs. 21% accurate; χ^2^ = 4.8; *p* < 0.05), and concreteness (concrete vs. abstract 57 vs. 33% accurate; χ^2^ = 6.8; *p* < 0.05). Spelling was significantly influenced by word-length: 78% correct on 4-letter words, 57% on 5-letter words, 50% on 6-letter words, 36% on 7-letter words, and 28% on 8-letter words (e.g., 4-letter vs. 5, 6, 7, 8 letters; *p* < 0.05). There was no effect of regularity.

Errors in spelling to dictation were mostly phonologically implausible nonword errors (e.g., parent → parpe) and some unrelated word errors (palace → pea). His 0% accuracy on nonword errors indicates impairment at the level of sublexical mechanisms for phoneme-grapheme conversion (PGC), although he was also impaired in access to written word forms. His word length effect can be explained by greater opportunity to err on longer words in his attempts to rely on (impaired) PGC. We decided to treat sublexical spelling—PGC—as a first step, to give him some rules he could rely on to at least produce a plausible spelling.

SMY's written picture naming performance was assessed using the Philadelphia Naming Test (PNT, Roach et al., 1996) to examine generalization from spelling-dictation to written picture naming. SMY's performance on the written picture-naming task was impaired. He scored 121/175 on the PNT prior to treatment. Errors in written naming were predominantly phonologically implausible nonword errors: 63% (candle → calc), some semantic errors: 13% (hose → cable), some unrelated word errors: 13% (mustache → mustang), and some no responses: 11%.

## Procedure

### tDCS treatment

We used a double-blind, within-subject crossover trial design, with random order of treatments. There were two experimental conditions: “right cerebellar tDCS + behavioral (spelling) treatment” and “sham tDCS + behavioral treatment.” Each condition consisted of 15 consecutive training sessions, 3–5 per week, separated by 2 months. Evaluation took place before, immediately after, and 2 months post-treatment for each condition (Tsapkini et al., 2014). SMY was randomized to the “sham” condition first followed by the “tDCS” condition. tDCS was administered for the first 20 min of the 45-min treatment session of behavioral spelling treatment. tDCS was delivered at a constant current of 2 mA for 20 min via two 25 cm^2^ saline soaked sponge electrodes using a ActivaDose II stimulator (ActiveTec Inc., Salt Lake City, Utah). The anode was centered on the right cerebellum (1 cm under, and 4 cm lateral to the inion: Pope and Miall, 2012) and the cathode was placed on the right deltoid muscle. Sham tDCS was applied using the same electrode configuration, but current intensity was ramped down to zero after 30 s (Gandiga et al., 2006).

### Behavioral spelling treatment

We employed a spelling treatment protocol previously described (Tsapkini et al., 2014) that specifically targeted PGC in spelling to dictation. We selected 80 words from the Johns Hopkins Dysgraphia Battery that SMY misspelled. Words were divided into two sets: trained words (*n* = 40), practiced during treatment (sham and tDCS), and untrained words (*n* = 40), only tested prior to the start of treatment, end of treatment, and 2 months post-treatment. Stimuli in both sets were matched for lexical frequency, letter length, and concreteness. Words were 4–8 letters long and consisted of nouns, verbs, and adjectives. For each treatment condition (sham and tDCS), we compared the correct responses (1) pre-treatment and immediately after treatment, (2) pre-treatment and 2 months post-treatment on each stimulus type (trained words, untrained words, or written picture naming) with McNemar's test for correlated responses (see Table 1).

**Table 1:** **Raw scores for trained words, untrained words, and written picture naming prior to the start of treatment, immediately after treatment and 2 months post-treatment for each condition**.

**Task**	**Sham**			**tDCS**		
	**Pre-treatment**	**Immediately after**	**2 months post-treatment**	**Pre-treatment**	**Immediately after**	**2 months post-treatment**
Trained words	0/40	21/40 (*p* < 0.0001)	13/40 (*p* < 0.0001)	13/40	39/40 (*p* < 0.0001)	39/40 (*p* < 0.0001)
Untrained words	0/40	11/40 (*p* = 0.0026)	11/40 (*p* = 0.013)	11/40	33/40 (*p* < 0.0001)	36/40 (*p* < 0.0001)
Written naming	121/175	121/175 (*p* = 0.75)	122/175 (*p* = 0.76)	122/175	143/175 (*p* = 0.0003)	145/175 (*p* = 0.0004)

*McNemar's test results (two-tailed, p-value) comparing the correct responses between (1) pre-treatment and immediately after treatment, (2) pre-treatment and 2 months post-treatment, on each stimulus type (trained word, untrained word, or written naming) are shown in italics. Shading indicates significant improvement*.

The behavioral spelling treatment consisted of training PGC in the context of each dictated word practiced. For each trained word, SMY was asked to point to the letter corresponding to each phoneme (from a set of letters). If he was correct, he was reinforced. If he was incorrect, the clinician pointed to the correct letter. Then SMY was asked to write the letter(s) corresponding to a particular phoneme for the trained word. If he was incorrect, the clinician wrote the correct letter. Finally, SMY was explicitly instructed in PGC for all letter-sounds of the word. Each session consisted of teaching the PGC of five trained words; when SMY met criteria (90% accuracy across three sessions), new words were introduced. It should be noted that SMY did not receive any other therapy except for support groups, including during follow-up periods.

### Modeling current flow in the right cerebellum

**Figure 2:**
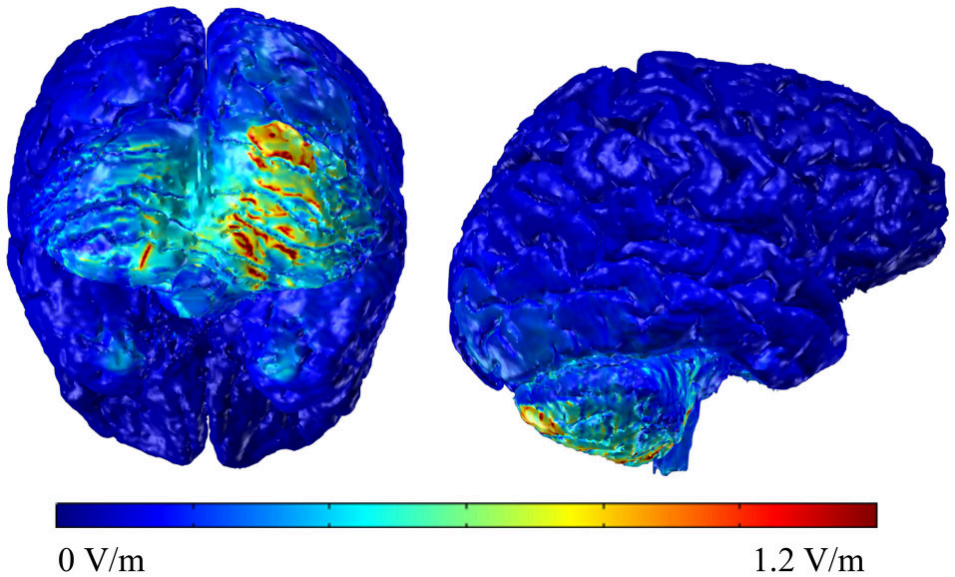
**Back and lateral views of the modeling data of the electric field distributions below the stimulating electrode on the right cerebellum**.

To understand the electric field distribution of right cerebellar tDCS, we completed a modeling study. One high resolution T1 MRI-scan (1 mm^3^ voxels) of a healthy control extending between the c7 vertebra and the vertex was segmented into 11 tissue compartments using automated algorithm and manual segmentation techniques using ScanIP (Simpleware) as previously described (Datta et al., 2009). Specific conductivity values were assigned to the individual tissue compartments. The MRI-based Finite Element Method (FEM) models were generated using COMSOL Multiphysics to predict current flow in volume conductor physics studies involving two 5 × 5 cm sponge pad electrodes. A 2 mA stimulation boundary condition was applied to the anode (right cerebellar cortex, 1 cm under, and 4 cm lateral to the inion) and a ground condition was applied to the cathode (right deltoid muscle). An electric isolation condition was applied to the remaining boundaries. Plots of the electric field profile (0–1.2 V/m) were displayed in a false color scale (blue-red) on a 3D rendering of the brain. The results indicate that the maximum electric field amplitude was generated in the right cerebellum with some spread to the left cerebellum but without spread to adjacent occipital cortex or other cortical areas (see Figure 2).

### MRI acquisition

Resting state fMRI was acquired twice: prior to the start of the study and 2 months after the completion of the study (6 month interval between scans). Scans were acquired on a 3-T Philips Achieva MRI scanner with a 32-channel head coil. Resting state images were acquired using EPI and the following scan parameters: TR = 2000 ms, TE = 30 ms, flip-angle = 90, matrix = 64 × 64, FOV = 240 × 240 mm, 35 3 mm parallel axial slices covering the whole brain, 210 volumes. High resolution 3D MPRAGE was acquired in the sagittal plan utilizing a multishot, turbo field echo pulse sequence and the following scan parameters: TR = 6800 ms, TE = 31 ms, matrix = 256 × 256, FOV = 256 × 240 × 240 mm, 170 1 mm slices covering the whole brain. One normal control (59/Female) was scanned longitudinally using the same scan interval as SMY.

Changes in connectivity were examined between the right cerebellum and peri-lesional language regions of interest (ROIs) in the LH and RH. Only non-lesioned regions were included in the ROI. The ROI included: superior frontal gyrus (SFG), superior frontal gyrus_prefrontal cortex (SFG_PFC), middle frontal gyrus/dorsolateral prefrontal cortex (MFG_DLPC), middle temporal gyrus pole (MTG_pole), inferior temporal gyrus (ITG), fusiform gyrus (FG), left and right cerebellum. For a full description of resting state-processing steps see Sebastian et al. (2016a). Briefly, the structural scan was segmented using the multi-atlas mapping and parcellation approach, co-registered to the motion and slice timing (SPM8) corrected resting state dynamics. Time courses were extracted from the specific ROIs, which were regressed for physiological nuisance by applying CompCor (Behzadi et al., 2007). From the “nuisance-corrected” time courses we obtained the parcel-by-parcel correlation matrices, z-transformed by Fisher's method. The whole procedure was performed automatically in BrainGPS (Li et al., 2015).

## Results

### Treatment data

SMY showed an overall improvement in spelling with a notable increase in writing speed. Trained words were the same for the sham and tDCS conditions. However, out of the 40 trained words, SMY reached criterion for only 17 trained words during the sham condition. On an average, he required 4.5 (range: 3–7) sessions to reach criterion for each trained word. SMY reached criterion more quickly during the tDCS condition. He was able to reach criterion in 3.2 sessions (range: 3–4).

SMY correctly spelled to dictation 21/40 trained words and 11/40 untrained words after sham treatment, whereas he correctly spelled to dictation 39/40 trained words and 33/40 untrained words after tDCS treatment. Therefore, both sham and tDCS treatments were effective for trained and untrained words immediately post-treatment, but there was significantly greater improvement with tDCS than with sham for both trained and untrained words (trained words: χ^2^ = 14.77, *p* = 0.0001; untrained words: χ^2^ = 15.758, *p* < 0.0001). At 2 months post-treatment, SMY showed maintenance of learned PGC rules in spelling to dictation on trained and untrained words in both tDCS and sham conditions; however, significantly greater maintenance was noted in the tDCS condition compared to the sham condition (trained words: χ^2^ = 26.97, *p* < 0.0001; untrained words: χ^2^ = 23.22, *p* < 0.0001). In addition, generalization from spelling to dictation to written picture naming was noted only in the tDCS condition.

SMY showed a notable change in spelling error types post-treatment, especially after the tDCS condition. Before treatment, SMY's spelling to dictation was 0% correct (40/40 errors) for both trained and untrained words (trained words: 33/40 or 82.5% phonologically implausible nonword errors; 3/40 or 7.5% phonologically plausible nonword errors; 4/40 or 10% unrelated word errors, untrained words: 35/40 or 87.5% phonologically implausible nonword errors; 2/40 or 5% phonologically plausible nonword errors; 3/40 or 7.5% unrelated word errors). After sham treatment, SMY made 19 errors on the 40 trained words (15/19 or 79% phonologically implausible nonword errors; 1/19 or 5% phonologically plausible nonword errors; 3/19 or 16% unrelated word errors) and 29 errors on the 40 untrained words (26/29 or 90% phonologically implausible nonword errors; 1/29 or 3% phonologically plausible nonword errors; 2/29 or 7% unrelated word errors). After tDCS treatment, he made one error on the trained words and seven errors on the untrained words. All errors were phonologically plausible nonword errors (e.g., pigeon → peigon), and were thus more functional than pretreatment errors.

### Resting state data

**Figure 3:**
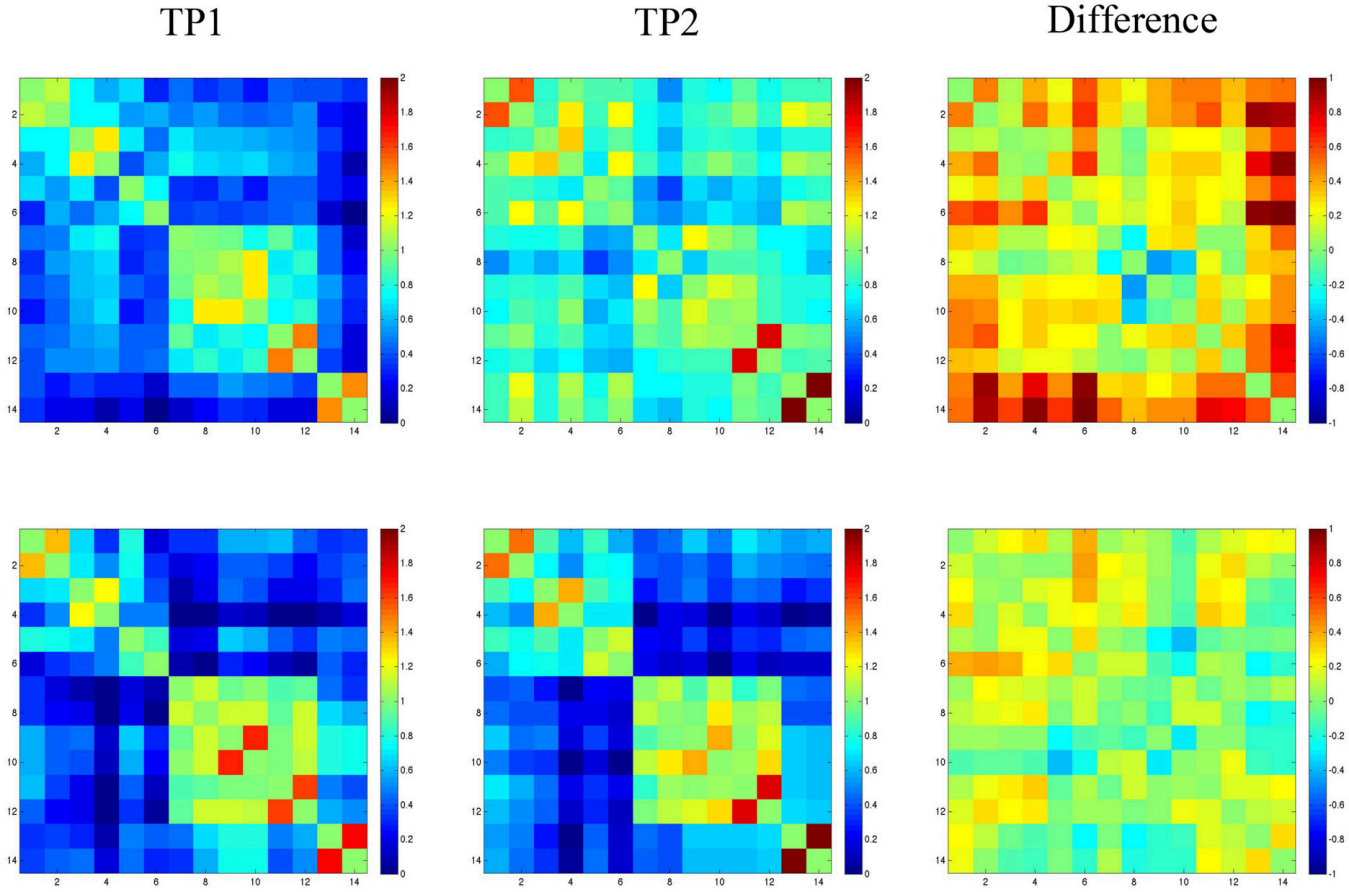
**Fisher-transformed correlation matrix for the resting state data for SMY (top panel) at time point 1 (TP1: prior to the start of treatment) and time point 2 (TP2: 2-months follow up time point)**. Control participant's data is shown in the **bottom panel**. Difference map shows the difference in correlation between the scan for the resting state data. Correlations were assessed across 14 ROIs. Regions are labeled as numbers corresponding to the left and right superior frontal gyrus (SFG; region 1 and 2), superior frontal gyrus_prefrontal cortex (SFG_PFC; region 3 and 4), middle frontal gyrus dorsolateral prefrontal cortex (MFG_DLPC; region 5 and 6), middle temporal gyrus pole (MTG_pole; region 7 and 8), inferior temporal gyrus (ITG; region 9 and 10), fusiform gyrus (FG; region 11 and 12), and cerebellum (region 13 and 14).

Resting state functional connectivity analysis data for SMY and the normal control are shown in Figure 3. For SMY, pre-treatment, weak correlations (connectivity) were noted between the right cerebellum and the left and right hemisphere ROIs, and also between the LH and RH ROIs (indicated by dark blue on the graph in Figure 3). For example, SMY showed weak connectivity (low z scores) between the right cerebellum and the left MFG_DLPC before treatment (dark blue color, 1st column 5th cell from bottom left). At 2 months post completion of treatment, connectivity was higher between the right cerebellum and ROIs in the LH and RH and also between the LH and RH ROIs (light blue and green colors). The difference map between pre-treatment resting state connectivity and 2 months post-treatment connectivity shows stronger correlations between the right cerebellum and the LH and RH ROIs (red, orange, and yellow). The control participant showed very similar connectivity at both the time points between (1) the right cerebellum and the LH and RH ROIs and (2) LH and RH ROIs. Difference map did not indicate a marked difference in z scores between time point 1 and 2.

## Discussion

This report is, to our knowledge, the first to use cerebellar neuromodulation to augment spelling recovery in an individual with large bilateral chronic strokes. This case study illustrated the potential usefulness of novel electrode placement for tDCS augmentation of language therapy in chronic post-stroke aphasia. Results suggest that anodal tDCS of the right cerebellum coupled with behavioral therapy is more effective than behavioral therapy alone in improving spelling to dictation. Furthermore, generalization to written picture naming was facilitated by tDCS, not sham. Finally, the resting state functional connectivity data indicate that improvement in spelling is accompanied by an increase in cerebro-cerebellar network connectivity.

We found robust improvement in SMY's spelling skills for both trained and untrained words especially after tDCS. Robust effects could be due to the duration and intensity of treatment (15 sessions, 3–5 per week). Alternatively, long-term stimulation may have induced long-term potentiation of neurons that may have lowered the threshold of neuronal excitability and subsequent modification in synaptic connectivity in the areas applied (e.g., Fritsch et al., 2010). In addition, cerebellum is a critical region involved in skill learning and repeated tDCS along with behavioral therapy might have facilitated the learning of writing skills and/or compensatory strategies. Facilitation of skill learning by cerebellar tDCS has been reported earlier. For example, Galea et al. (2011) showed that anodal tDCS applied over the cerebellum during reaching adaptation task facilitated learning. Similarly, Ferrucci et al. (2013) showed that anodal tDCS applied over the cerebellum during a procedural learning task facilitated implicit learning.

Although, right cerebellum is not traditionally considered to be associated with spelling, we show that cerebellar tDCS along with spelling therapy resulted in significant improvement in SMY's spelling abilities. Given that SMY sustained two large strokes involving the left and right hemispheres, tDCS may have enhanced changes in neuroplasticity resulting in modification of networks underlying spelling.

## Conclusions

This study, although preliminary, yielded interesting findings and promising avenues for further studies on cerebellar tDCS in post-stroke aphasia. Limitations include that this was a case study. In addition, tDCS followed sham in this experimental design and thus any extra benefits of tDCS might in fact be the benefits of having a second treatment period after already having had the first treatment period and time for consolidation. Cerebellar tDCS may not have comparable beneficial effects in patients with unilateral LH lesions. Also, resting state data were not acquired before and after completion of each treatment condition (i.e., sham and tDCS). Therefore, it is not possible to disentangle the network connectivity changes associated with tDCS vs. behavioral treatment.

## Author contributions

All authors listed, have made substantial, direct and intellectual contribution to the work, and approved it for publication. AH, RS, KT, and PC were involved in conception and design, analysis and interpretation of data. RS, SS, CL, AM, CD, and DT performed language evaluation, tDCS treatment, and or neuroimaging data acquisition. AM and MB performed computational modeling and interpretation. AF performed neuroimaging analysis and interpretation. RS, AH, KT, DT, and PC drafted the article and revised it critically for important intellectual content.

### Conflict of interest statement

The authors declare that the research was conducted in the absence of any commercial or financial relationships that could be construed as a potential conflict of interest.
