# Supplementary material for: Cerebellar tDCS: A Novel Approach to Augment Language Treatment Post-stroke
Source: Front Hum Neurosci. 2017 Jan 12;10:695. doi: 10.3389/fnhum.2016.00695 (PMC5226957; doi:10.3389/fnhum.2016.00695)
Supplement: Supplementary file 1 [file Table1.PDF]

S1. SMY's performance on the Johns Hopkins dysgraphia battery

Parts of Speech	
Open class words	22/84
Nouns	6/28
Verbs	10/28
Adjectives	3/28
Function words	3/20
Nonwords	0/34
Concreteness	
Concrete words	12/21
Abstract words	7/21
Word Length	
4-letter	11/14
5-letter	8/14
6-letter	7/14
7-letter	5/14
8-letter	4 /14
Probability (PG regularity)	
High Probability	13/20
High Frequency	7/10
Low Frequency	6/10
Low Probability	12/30
High frequency	8/15
Low frequency	8/15
